# Mouthparts and Alimentary Tract of Flower-Visiting Monkey Beetles (Coleoptera: Scarabaeoidea: Hopliini): Insights into Feeding Preferences

**DOI:** 10.3390/insects16090985

**Published:** 2025-09-21

**Authors:** Michael Neulinger, Florian Karolyi, Jonathan F. Colville, Myriam E. Widmann, Jonas Kristl, Harald W. Krenn

**Affiliations:** 1 Unit Integrative Zoology, Department of Evolutionary Biology, University of Vienna, Djerassiplatz 1, 1030 Vienna, Austria; mike2700410@gmail.com (M.N.); florian.karolyi@univie.ac.at (F.K.); myriam.widmann@hotmail.com (M.E.W.); jonas.kristl@liwest.at (J.K.); 2 Centre for Statistics in Ecology, the Environment and Conservation (SEEC), Department of Statistical Sciences, University of Cape Town, North Ln, Rondebosch, Cape Town 7700, South Africa; jonathan.colville@gmail.com

**Keywords:** feeding organs, gut, flower-visiting insects, morphology, µCT imaging, Coleoptera, floral tissue, pollen, nectar

## Abstract

Monkey beetles (Hopliini, Scarabaeoidea) are a species-rich group of flower-visiting insects that are specialized to feed on floral tissue, pollen and/or nectar. We studied ten South African species, examining morphological features associated with their specific feeding preferences. This is the first attempt in a limited number of beetle species to comparatively investigate both the mouthparts and the alimentary tract in relation to ingested food. Using light microscopy, we found cutting edges on the mandibles and galea teeth in flower tissue-feeding species. Pollen feeders have numerous bristles on the maxillae and a prominent mola on the mandibles that are likely used for gathering and grinding pollen. The elongate heads and mouthparts of the nectar feeders are considered an adaptation that enable these species to mop up nectar while probing flowers. Using µCT imaging and reconstructions of the entire alimentary tract, our morphometric results suggest that food preferences are not related to total relative gut length, although the ratio of foregut to body length was greater in pollen- and nectar-feeding monkey beetles than in floral tissue-feeders. The midgut of females tends to be longer relative to body size compared to males. Our work serves as a basis for generating hypotheses for future research that includes gut morphology in flower-visiting insects.

## Simple Summary

We studied the feeding organs and digestive tracts of ten species of flower-visiting monkey beetles (Hopliini, Scarabaeoidea) in relation to their feeding preferences. Using light microscopy, we demonstrate that beetles that feed on floral tissues possess cutting edges and teeth on their mandibles and maxillae. Pollen-feeding beetles have setose mouthparts equipped with grinding structures, whereas nectar feeders are characterized by elongated heads and feeding organs. Comparisons of the digestive tracts using µCT techniques suggests that the foregut of pollen- and nectar-feeding beetles is longer than that of floral tissue-feeders. The midgut of female beetles tends to be longer than that of males.

## 1. Introduction

Out of over one million Hexapoda, more than 440,000 described species belong to the Coleoptera [1], which makes beetles the most species-rich taxon of insects. Given this considerable number, it is not surprising that a wide range of feeding strategies have evolved—including the use of floral resources—even though beetles are generally thought to possess unspecialised mouthparts [2]. Many flower-visiting beetles act as pollinators, with floral tissue, pollen and nectar offered as food rewards to attract these insects as pollen vectors [3]. In contrast to Coleoptera, many anthophilous insects show remarkable morphological adaptations of the feeding organs to consume floral resources [2,4,5]. However, nearly all beetles, including flower-visiting species, possess rather unspecialised prognathous biting-chewing mouthparts, which are composed of paired mandibles and maxillae as well as the lip-like labrum and labium [2,6]. Although the biting–chewing functional type of mouthparts seen in beetles is often regarded to be unspecialized compared to the elongated and highly modified feeding organs of other flower-visiting insects such as bees and butterflies [5], anthophilous beetles, including monkey beetles [7], possess subtle morphological adaptations to petal-, pollen- and nectar-feeding [8,9,10]. Observations in monkey beetles showed that brushes of the maxillae are well suited for sweeping up pollen and mopping up nectar by semi-circular movements of the mouthparts [11].

In insects, ingested food is processed and absorbed by the digestive tract which possesses specialized compartments with various outgrowths, each with different tasks [12,13]. The ectodermal foregut can be divided into pharynx, oesophagus, crop and proventriculus. The pharynx is easily recognized by its position in relation to the brain inside the head. Many dilator muscles attach to this expendable region and act as antagonists to the muscular sheath of longitudinal and circular muscle fibres surrounding the entire foregut. The crop is the most prominent structure of the foregut, and its volume is related to the storage capacity of this organ. The proventriculus, located at the transition of foregut to midgut, is usually the most complex structure of the entire alimentary tract. In adult, solid food feeding insects, it is often equipped with cuticular teeth or plates, whereas many nectar feeders have lost these structures [13,14]. From the proventriculus, food is passed through a valve into the endodermal midgut. Compared to the foregut, the muscular sheath of the midgut is less developed. Outgrowths of the anterior midgut, called gastric caeca, are common and can vary in number. In pollen-feeding scarab beetles, it was shown that pollen digestion starts in the anterior midgut [11]. The most striking outgrowths of the alimentary tract are the Malpighian tubes, of which there are four to six in beetles, and excretory diverticula located in the anterior end of the hindgut [13,14]. The ectodermal hindgut is divided into the anterior and posterior intestine, the former further divided into the ileum and the colon. The posterior intestine can be dilated into a rectal sac, then gradually narrowing down, which attaches to the anus [13,14]. Many beetle species share a basic alimentary tract morphology with various shapes and sizes of the individual gut regions [15,16,17,18,19,20,21,22,23,24]. The gut of beetles can vary in length, depending on the type of food they consume, with herbivorous species having longer midguts in relation to body size compared to carnivorous species [15]. In addition, larval beetles feed on different types of plant tissues than adults: in the Scarabaeoidea, larvae consume rotting plant material while adult Scarabaeoidea are regular flower visitors which may take up floral tissue, pollen and/or nectar [3,11,25]. In pollen-feeding Coleoptera, most of the pollen grains were found to be empty in the hind gut where the exines are intact but collapsed, indicating digestion and loss of the pollen content [11].

Monkey beetles (Hopliini) show their greatest diversity in the Greater Cape Floristic Region of South Africa where they are known to be important pollinators for many plants belonging to different families [26,27,28,29]. Flowers serve as food sources for adult monkey beetles and as mating sites. Based on their colour preference and feeding strategies, monkey beetles were placed in two broad putative feeding guilds [29]. In the first “non-embedding” guild, species of genera such as *Anisonyx*, *Lepithrix* and *Peritrichia*, were hypothesised to feed on pollen and possibly nectar. Members of the second “embedding” feeding guild, including beetles from genera such as *Gymnoloma*, *Heterochelus*, *Pachycnema* and *Scelophysa*, dig into the flowers. Based on this behaviour they are hypothesised to feed on floral tissue, including ovules and pollen. A previous study refined these feeding guilds based on mouthpart morphology and defined three different feeding groups: (1) floral/plant tissue feeders; (2) pollen feeders, and (3) nectar feeding monkey beetles; the latter two show intermediate traits suggesting adaptations to utilizing both pollen and nectar [7].

Interestingly, no unambiguous matching between monkey beetle mouthpart morphology and their feeding behaviour was found [7]. For example, the authors noted contrasting findings between mouthpart morphology, gut contents and the previously defined guild structure [29]. These results suggest that these mismatches require further detailed analyses of the digestive tract to confirm if hypothesised feeding preferences are related to morphological traits and gut content. No comparative study currently exists that is focused on the morphology and biometry of the digestive tract in relation to various floral food preferences. The present study aims to combine examination of the mouthparts, the alimentary tract using µCT techniques and the gut content to investigate if the food preferences of monkey beetles are related to the structures of the feeding organs and the gut morphology. This study is the first to incorporate the alimentary tract into analyses of eco-morphological adaptations to specific food sources in flower-visiting insects.

## 2. Materials and Methods

### 2.1. Sample Acquisition

All studied specimens of monkey beetles (Table 1) were collected from flowers in the Western and Northern Cape Province of South Africa, between August and September 2016 and 2017. Beetles were fixed in either FAA (formalin-aceto-alcohol) or 70% ethanol in the field, and, subsequently, all samples were stored in 70% ethanol.

### 2.2. Gut Content Analysis

The gut content of each individual species was determined by conventional dissection or from previous publications [7]. If floral tissue with little or no pollen grains were found, then specimens were considered as floral tissue feeder. Similarly, if the gut content of a specimen contained mainly pollen with little or no floral tissue, it was classified as a pollen feeder. Specimens with little or no pollen and floral tissue in their gut were classified as nectar feeders. Extracted food was embedded in glycerine on a microscope slide and covered with a cover glass. Each slide was analysed with a Labophot-2 microscope (Nikon Corporation, Tokyo, Japan) and the gut content of each species was recorded.

### 2.3. Light Microscopical Study of Mouthpart Morphology

Mouthparts were dissected under a stereo microscope (Leica MZ 125, Leica Camera AG, Wetzlar, Germany) and stored in 70% ethanol. A set of mouthparts from each species was submerged in polyvinyl lactophenol and covered with a cover slip. Microscopic slides were placed in a drying cabinet Binder FED-53 (Fisher Scientific GmbH, Schwerte, Germany) at 50 °C for several days. Finally, the edges of the cover slips were sealed with transparent nail polish. Specimen slides were photographed using a stereo microscope Nikon SMZ 25, equipped with a Nikon DS-Ri2 camera (Nikon Corporation, Tokyo, Japan), outfitted with NIS-Elements BR software version 5.01.00 [30]. Extended depth of field images were taken of the individual mouthparts of all species. All images were enhanced for clarity and visual fidelity using Adobe Photoshop version 22.4.2 [31].

### 2.4. Micro Computer Tomography and 3D-Imaging

One male and one female from each species were selected for micro-CT scanning. In case only one sex of a species was available, two beetles from the same sex were chosen (Table 1). The protocol for staining of micro-CT insect samples by Metscher [32] was followed. Beetles chosen for micro-CT scanning were placed in 80% ethanol for approximately 24 h, transferred to 90% ethanol for 24 h, and finally stored in 100% ethanol before staining in I2E (1% iodine metal dissolved in 100% ethanol) for 7 to 14 days, depending on body size. All samples were placed on a rocker (VWR Advanced Digital Shaker, Radnor, PA, United States of America) during the staining process. After staining, samples were washed in 100% ethanol to remove excess I2E from the tissue and stored in 100% ethanol. Specimens were placed in a 1000 µL plastic pipette tip (Eppendorf SE, Hamburg, Germany), heat sealed at the tip filled with 100% ethanol and subsequently sealed with Parafilm laboratory film (Bemis Company Inc., Neenah, WI, United States of America). Micro-CT scans of the beetles were produced at the University of Veterinary Medicine, Vienna, using a µCT35 (SCANCO Medical AG, Brüttisellen, Switzerland) with a voxel size of 6 and 3.5 µm, 70 kV peak intensity and an AL 0.5 mm filter. Additional scans were produced at the Department for Evolutionary Biology, University of Vienna, using a SKYSCAN 1272 (Bruker Corporation, Billerica, MA, United States of America). The latter samples were scanned with an image voxel size ranging from 3 to 6 µm and 60 kV peak intensity and an AL 0.5 mm filter.

#### 2.4.1. D-Reconstruction

Assignment of the correct voxel size and editing of the µCT-image stacks was performed with the 3D software Amira version 6.4 [33]. The alimentary tract of all species was marked for uniform segmentation based on morphological features. The anterior end of the alimentary tract was defined by an imaginary line drawn between the clypeus base and labium base. The anterior pharynx and the posterior end of the crop were chosen as the anterior and posterior ends of the foregut. The anterior part of the ventriculus marked the beginning of the midgut, and the confluence of the Malpighian tubes marked the end of the midgut and the beginning of the anterior hindgut. Posterior areas of the hindgut and alimentary tract were defined by the opening of the rear end of the rectum into the genital chamber.

#### 2.4.2. Body Length Measurement

Based on a volume rendering with an Ortho Slice in Amira, a line set with the “B-Spline” was created. This allowed manual placement of points on a selected image plane, displayed by the command “Ortho Slice” and its function to slice the volume rendering from three different axes based on micro-CT images. Using the line editor of the “B-Spline” command, a point was placed at the anterior end of the body, while the second point was placed at the posterior end of the abdomen. This “B-Spline” line was converted to a spatial graph with the command “Line Set To Spatial Graph”.

#### 2.4.3. Alimentary Tract Length Measurement

A specific sequence of commands was followed for accurate length measurements in Amira. First, the command “Arithmetic” was used to recolour the 8-bit grey scale material (“Gut”) of each specimen’s label file with the line “(A = 2) × 255” to the highest white colour value. Afterwards, “Resample” scaled down the voxels of the material by a factor of eight. Finally, a material’s voxel skeleton of each label file was created with “Distance-Ordered Thinner”. This resulted in a line of individual dots, which were linked together by “Trace Lines” to a continuous line through the selected material. Smoothing was performed on the spatial graph with the command “Smooth Line Set”. The division of the spatial graph was accomplished by converting it to a line set with the command “Spatial Graph To Line Set”. Subsequently, by entering the line set editor, the graph was split, resulting in three distinct lines for the three main gut sections. Every line of each section was converted back to a spatial graph by the command “Line Set To Spatial Graph”.

### 2.5. Image Creation

Internal gut surface renderings were created with the tool “Generate Surface” without smoothing the surface. Subsequently, a uniform 3D-gut surface was achieved with “Smooth Surface” and the process of face reduction with the surface editor. Images were generated with the snapshot tool in Amira. The surfaces of the alimentary tract of each beetle were set to 65% transparency and FrontFace lightning in the 3D-viewer and were combined with their corresponding spatial graph (visualized as tube, size 20). Finally, image scale bars were adjusted with ImageJ version 1.46r [34].

### 2.6. Data Visualization

The software Excel version 2410 [35] was chosen for the visual representation of data in the form of Box and Whisker plots [36]. All measurements of the length of the digestive tract of each beetle were expressed as ratios of gut to body length.

## 3. Results

### 3.1. Field Observations of Feeding Monkey Beetles

**Figure 1:**
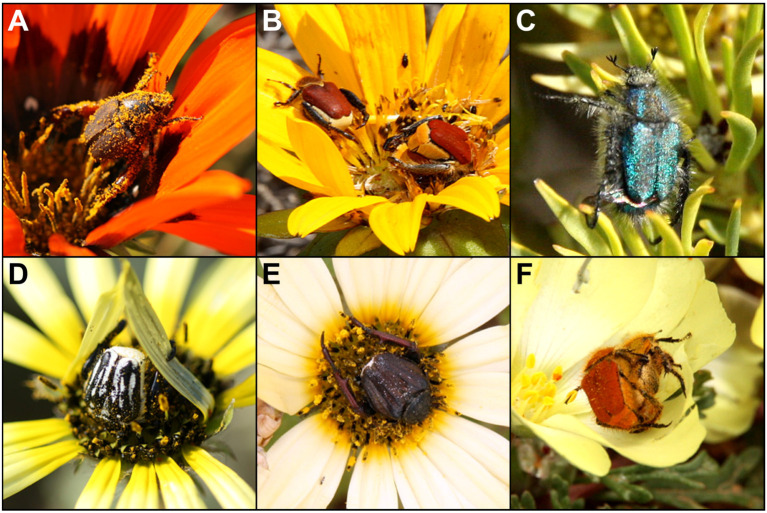
Selected studied species of monkey beetles on their host plants; (**A**) *H. detritus* on *Gazania rigida* (Burm.f.) Roessler, 1959; (**B**) *S. strandfonteinensis* on *Didelta carnosa* (L.f.) Aiton, 1789; (**C**) *A. ditus* on *Leucadendron salignum* P.J. Bergius, 1766; (**D**) *P. crassipes* on *Arctotheca calendula* (L.) Levyns, 1942; (**E**) *D. squamosum* on *Arctotheca calendula*; (**F**) *L. colvillei* on *Grielum humifusum* Thunb., 1800.

The predicted food sources of species were assigned according to field observations. The species *Heterochelus detritus* (Figure 1A), *S. strandfonteinensis* (Figure 1B), *P. crassipes* (Figure 1D) and *D. squamosum* (Figure 1E) were predicted to feed from the disk floret and/or receptacle, summarized as floral tissue. *Anisonyx ditus* (Figure 1C) and *A. ursus* were expected to take up nectar, while *A. inornatus*, *C. glenlyonensis*, *B. gifbergensis* and *L. colvillei* (Figure 1F) were presumed to be pollen-feeding beetles (Table 2).

### 3.2. Mouthpart Morphology and Food Type

**Figure 2:**
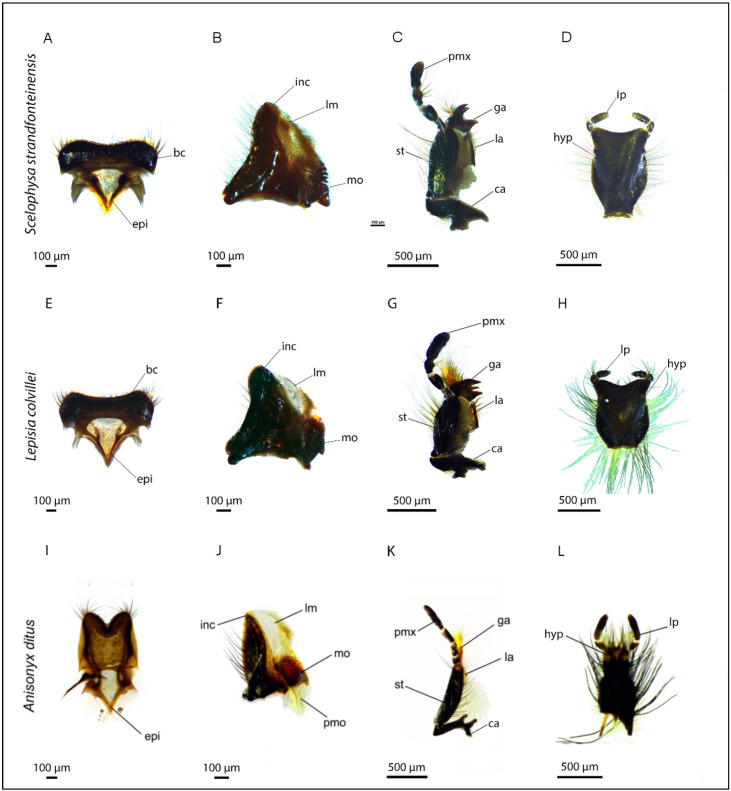
Mouthparts of three Hopliine beetles from different feeding groups; (**A**–**D**) floral-tissue feeding *S. strandfonteinensis*: (**A**) labrum, (**B**) mandible with strongly sclerotized lacinia mobilis and prominent mola, (**C**) maxilla with large apical teeth and short bristles, (**D**) labium; (**E**–**H**) pollen feeding *L. colvillei*: (**E**) labrum, (**F**) mandible with membranous lacinia mobilis and small mola, (**G**) maxilla with prominent apical teeth and long and dense bristles, (**H**) labium with long bristles; (**I**–**L**) nectar feeding *A. ditus*: (**I**) weaker sclerotized, elongated labrum, (**J**) mandible with large, membranous lacinia mobilis and reduced mola, (**K**) long and slender maxilla missing apical teeth, with reduced lacinia, (**L**) long and slender labium with prominent bristles; hair crest (bc), epipharynx (epi), incisivus (inc), lacinia mobilis (lm), mola (mo), postmola (pmo), cardo (ca), stipes (st), lacinia (la), galea (ga), palpus maxillaris (pmx), hypopharynx (hyp) and labial palpus (lp).

The mouthparts of all studied species were prognathous and appeared similar regardless of feeding preferences (Figure 2). Apart from size differences, the feeding organs of males and females showed no morphological differences. The labrum of all species was bilobed and beard bristles on the dorsal edges. The sclerotization was found to be weak in *A. ditus*, *A. ursus*, *D. squamosum*, and *H. detritus*. The labrum was slender and elongated in *A. ditus* (Figure 2I) and *A. ursus*, in contrast to *L. colvillei* and *S. strandfonteinensis*, which had a short, wide and sclerotized labrum (Figure 2A,E).

In all species, the mandibles were short and triangular and consisted of an inconspicuous incisivus, a mola and a soft lacinia mobilis with a postmola. The incisivus of the mandible showed a cutting edge in *H. detritus*, whereas in all other species it was found to be rounded. The mola showed rows of small teeth in all species (Figure 2B,F,J), except *H. detritus*. Across species, the lacinia mobilis differed in size and showed variation in shape and number of bristles. The maxillae differed in the shape of the cardo, stipes and galeae as well as the length of the palpus and number and length of the bristles (Figure 2C,G,K).

In *Anisonyx ditus* and *A. ursus*, all mouthparts were elongated and setous (Figure 2I–L). *Heterochelus detritus*, *S. strandfonteinensis*, *L. colvillei* and *B. gifbergensis* featured well-developed apical teeth on the galea (Figure 2C,G). Further, particularly long galea bristles were found in *D. squamosum* and *P. crassipes*.

The labium appeared rectangular, strongly sclerotized and ventrally covered with bristles (Figure 2D,H). The elongated labium of *A. ditus* (Figure 2L) and *A. ursus* bore numerous long bristles. Mouthpart food types were assigned to each studied species (Table 2).

### 3.3. General Gut Morphology

**Figure 3:**
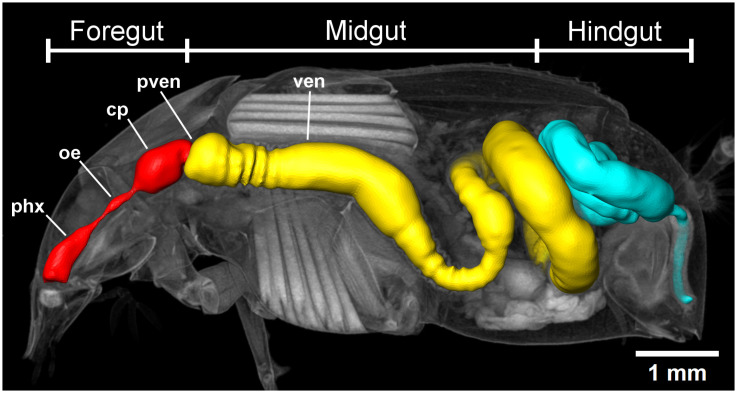
3D-reconstruction of the general alimentary tract morphology of a typical monkey beetle, with the three distinct gut regions and compartments using the example of *A. ditus;* sagital view; pharynx (phx), oesophagus (oe), crop (cp), proventriculus (pven), ventriculus (ven); foregut (red), midgut (yellow), hindgut (cyan).

The alimentary tract of monkey beetles is a tube-like organ extending through the body (Figure 3). The anterior opening of the alimentary tract is the buccal cavity in front of the pharynx, which continues posteriorly and gradually narrows into a short and slender oesophagus, which ventrally passes the brain. The oesophagus widens posteriorly into the crop, which in some species is balloon-shaped (Figure 3). The proventriculus is reduced to four inconspicuous folds protruding into the lumen of the crop, lacking any sclerotized structures like teeth or plates. The general appearance of the foregut is similar across all species, while the length and volume vary among species and between sexes. A constriction of the alimentary tract is often visible at the junction of crop and ventriculus, generally defined as the valve from foregut to midgut. The midgut is the most prominent section of the entire alimentary tract, being longer and more voluminous than all other parts of the gut (Figure 3). The anterior region is straight and characterized by circular folds of the inner intestinal wall visible in the three-dimensional reconstructions of the gut lumen. No midgut caeca or diverticula were found. The posterior region of the midgut is a smooth tube without internal structures. The midgut and posterior parts formed several loops in the abdomen (Figure 3). The shape and number of loops of the alimentary tract are variable among the species and between the sexes. The posterior end of the midgut is characterized by the origin of the four Malpighian tubes. The combined posterior sections ileum, colon and rectum form loops in the abdomen. The rectum extends vertically into the genital chamber where it opens with the anus (Figure 3).

### 3.4. Alimentary Tract in Feeding Groups

**Figure 4:**
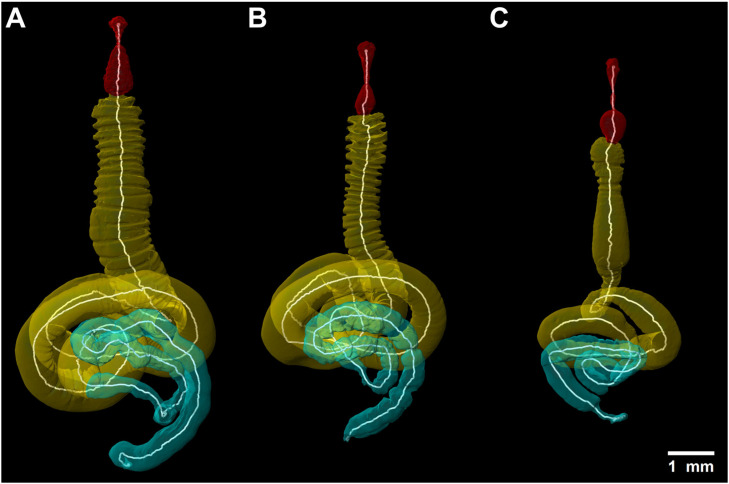
3D-reconstruction of the alimentary tract lumen of female floral tissue-feeding *S. strandfonteinensis* (**A**), pollen-feeding *L. colvillei* (**B**) and nectar-feeding *A. ditus* (**C**), in dorsal view; foregut (red), midgut (yellow), hindgut (cyan), with white measurement lines.

**Figure 5:**
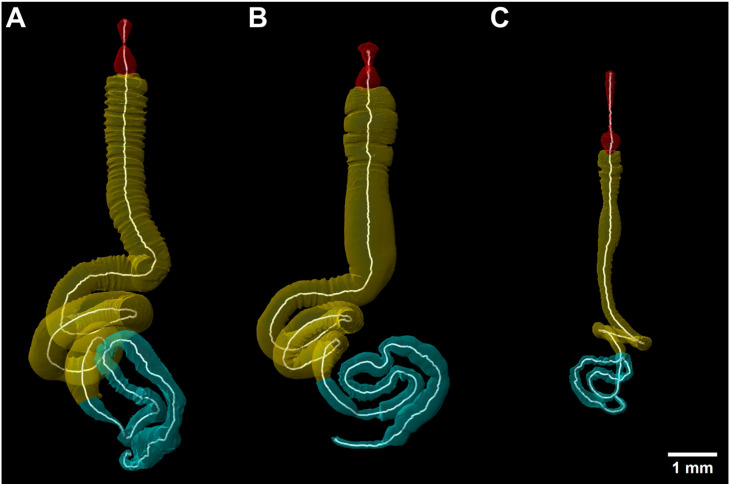
3D-reconstruction of the alimentary tract lumen of male floral tissue-feeding *S. strandfonteinensis* (**A**), pollen-feeding *L. colvillei* (**B**) and nectar-feeding *A. ditus* (**C**), in dorsal view; foregut (red), midgut (yellow), hindgut (cyan), with white measurement lines.

No specific anatomical features of the alimentary tract were found in any of the feeding groups. However, putative floral tissue and pollen feeders show more prominent infoldings of the anterior midgut than the nectar-feeding species (Figure 4 and Figure 5).

The posterior midgut of all feeding groups is characterized by conspicuous loops; a feature more prominent in female beetles (Figure 4A–C). Most of the supposed nectar feeders have an elongated head and pharynx, a crop with roundish looking features and fewer infoldings of the midgut, except in *A. ditus* where the midgut tissue appears smooth (Figure 4C and Figure 5C). Specimens of *L. colvillei* have a shorter oesophagus in comparison to other studied species of this guild, while the female specimen of *L. colvillei* exhibited a high degree of infolding of the anterior midgut tissue (Figure 4B and Figure 5B). However, the anterior midgut tissue of the male specimen of *L. colvillei* was defined by very low infolding (Figure 5B). The specimens of *S. strandfonteinensis* exhibit noticeable differences, such as a longer crop, and a greater number of coils in the midgut of the female specimen (Figure 4A and Figure 5A).

### 3.5. Gut Content

Gut content was visible on micro-CT images and could be found in most species. Food was often accumulated in the voluminous midgut and hindgut. Female guts especially contained large amounts of food while males stored much less, or food was not detectible. Floral tissue and plant fibres were discovered in the gut of *A. inornatus* and *S. strandfonteinensis*. In *A. ursus*, the gut contained a few pollen grains and an unidentified soft substance. In the genus *Beckhoplia* large amounts of pollen were visible in the midgut, but no plant fibres or floral tissue could be found (Table 2).

### 3.6. Morphometry of the Alimentary Tract

**Figure 6:**
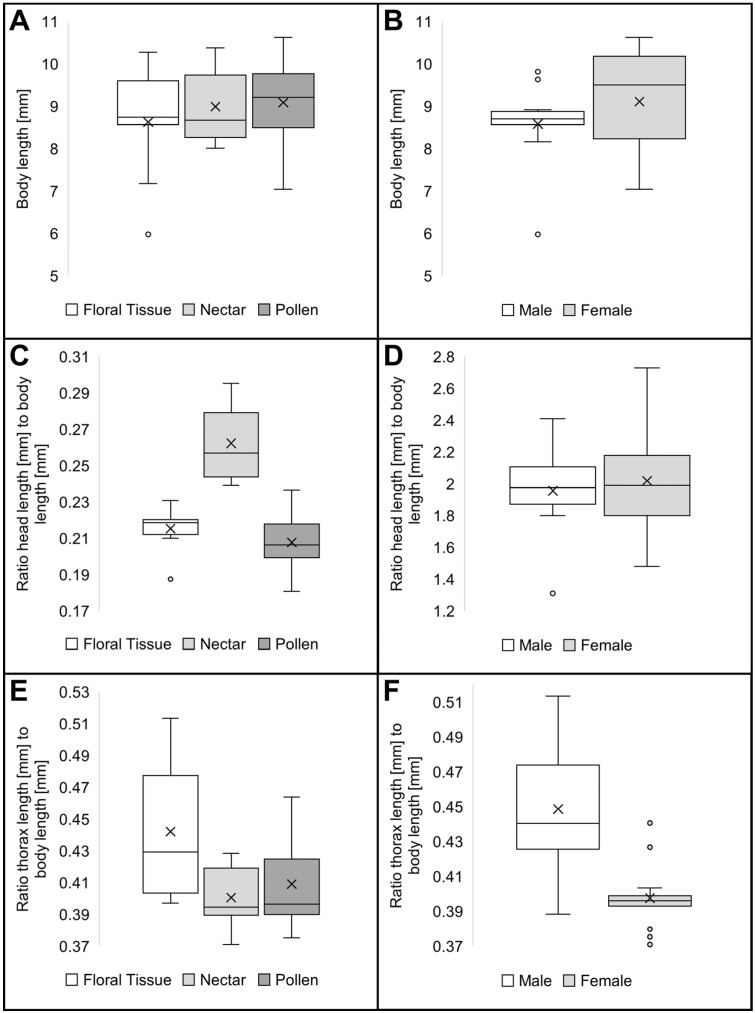
Morphometry of body length among the predicted feeding guilds and between male and female monkey beetle species; (**A**) total body length, floral tissue feeders (n = 9, SD = 1.26), nectar feeders (n = 6, SD = 0.90), pollen feeders (n = 8, SD = 1.07); (**B**) total body length in males (n = 10, SD = 0.99) and females (n = 13, SD = 1.18); (**C**) ratio head length to body length of floral tissue feeders (n = 9, SD = 0.01), nectar feeders (n = 6, SD = 0.02), and pollen feeders (n = 8, SD = 0.02); (**D**) ratio of head length to body length in male (n = 10, SD = 0.27), female (n = 13, SD = 0.34); (**E**) ratio of thorax length to body length of floral tissue feeders (n = 9, SD = 0.04), nectar feeders (n = 6, SD = 0.02), and pollen feeders (n = 8, SD = 0.03); (**F**) ratio of thorax length to body length in males (n = 10, SD = 0.04) and females (n = 13, SD = 0.02).

No obvious differences in body length exist in the feeding groups and sexes (Figure 6A,B). However, the ratio of head to body length is greater in suspected nectar-feeding species (Figure 6C), in contrast to the head length between sexes (Figure 6D). Additionally, the ratio of thorax length to body length appears to be larger in presumed floral tissue feeding species and male specimens (Figure 6E,F).

**Figure 7:**
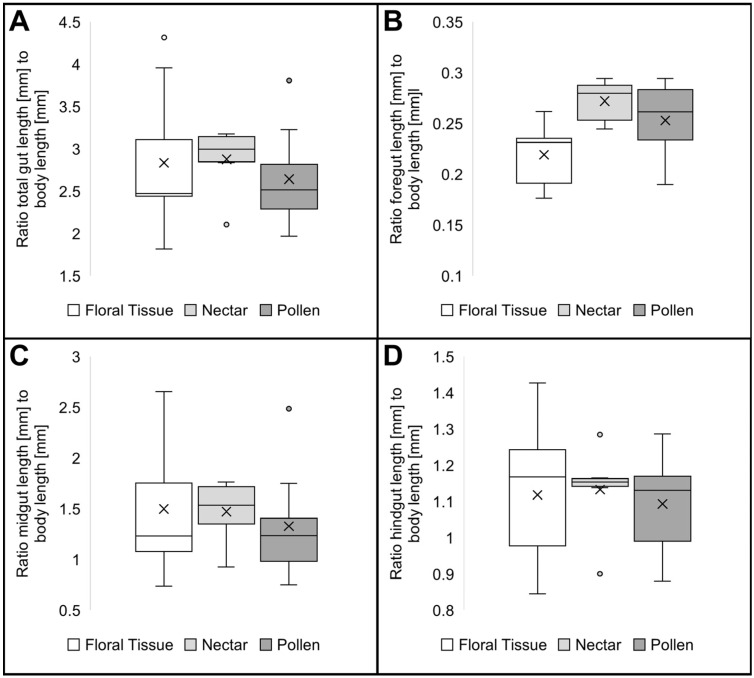
Comparison of gut length ratios between the predicted feeding guilds of monkey beetle species (Hopliini); (**A**) total gut length, floral tissue feeders (n = 9, SD = 0.77), nectar feeders (n = 6, SD = 0.37), pollen feeders (n = 8, SD = 0.57); (**B**) foregut length, floral tissue feeders (n = 9, SD = 0.03), nectar feeders (n = 6, SD = 0.02), pollen feeders (n = 8, SD = 0.04); (**C**) midgut length, floral tissue feeders (n = 9, SD = 0.63), nectar feeders (n = 6, SD = 0.29), pollen feeders (n = 8, SD = 0.52); (**D**) hindgut length, floral tissue feeders (n = 9, SD = 0.18), nectar feeders (n = 6, SD = 0.11), pollen feeders (n = 8, SD = 0.12).

The morphometric analysis revealed a trend to a greater relative foregut length in the suspected nectar and pollen feeding species (Figure 7B). The comparisons of the other sections of the alimentary tract revealed no obvious length differences of predicted feeding groups across the species studied (Figure 7A,C,D).

**Figure 8:**
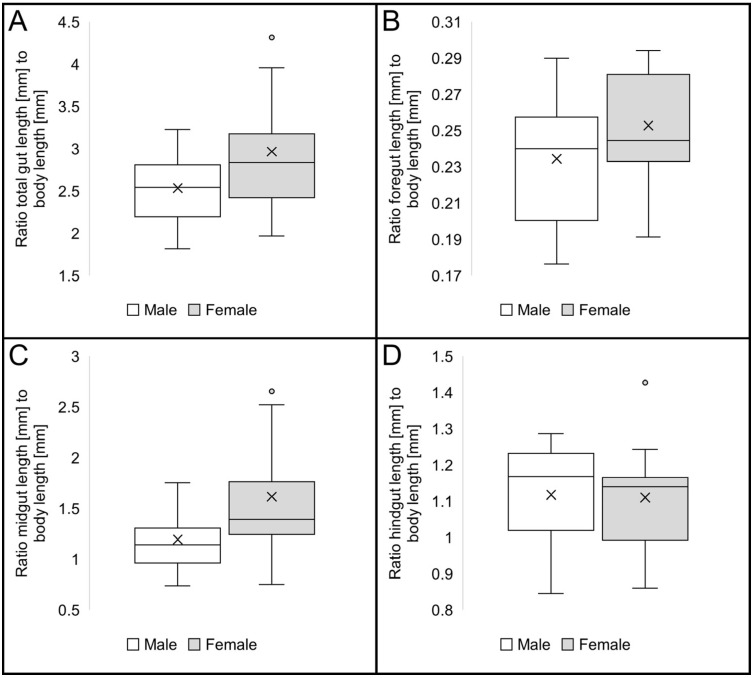
Comparison of gut length to body length ratios in male and female Hopliini; (**A**) total gut length, male (n = 10, SD = 0.43), female (n = 13, SD = 0.68); (**B**) foregut length, male (n = 10, SD = 0.04), female (n = 13, SD = 0.03); (**C**) midgut length, male (n = 10, SD = 0.32), female (n = 13, SD = 0.58); (**D**) hindgut length, male (n = 10, SD = 0.15), female (n = 13, SD = 0.15).

No obvious differences in the length of the gut or any of its sections could be detected between the sexes (Figure 8A–D). In general, the mid- and hindgut of female monkey beetles was more robust (Figure 4A–C), but not distinctly longer compared to males (Figure 8B–D).

The longest gut had a length of 44 mm, being 4.5 times longer than the body, recorded in a specimen of *S. strandfonteinensis*. The shortest total gut length of roughly 11 mm was recorded in a male beetle of *A. inornatus,* being only 1.8 times the body length (Table 3). The median of the ratio of total gut length to body size across all species is approximately 2.5 in males and approximately 2.8 in females (Figure 8A). The comparison of the gut sections reveals a trend of longer midguts in females (Figure 8C).

## 4. Discussion

### 4.1. Mouthpart Adaptations and Guild Structure

South African flower-visiting monkey beetles have previously been classified into feeding guilds based on observations of their behaviour on flowers [29] and more recently on their mouthpart structure [7]. This study is the first to examine the morphology of the mouthparts and alimentary tract of flower-visiting beetles simultaneously to identify features associated with their preferred diets. Our observations are consistent with previous findings that the morphology of the biting/chewing mouthparts of monkey beetles shows functional adaptations to feeding preferences [7]. Adaptations to floral tissue-feeding include cutting edges on the mandibles and teeth-like structures on the maxillae. The putative pollen feeders are equipped with numerous bristles and hairs that function like a brush to collect pollen, and the mandibles show a lobe-like lacinia mobilis and a prominent mola to transport and probably grind pollen grains. In contrast, suspected nectar-feeding monkey beetles have rather long and slender mouthparts without cutting edges on the mandibles. The slender and setous galeae are used to mop up nectar while probing flowers and the soft lacinia mobilis help to sweep nectar into the mouth. Forward pointing ligulae of the labium found in pollen- and nectar-feeders were hypothesized to be functional for harvesting pollen and nectar [7]. Many nectar-feeding insects have evolved elongated mouthparts and proboscises to extract nectar [5,37] because nectaries are often concealed in hard-to-reach areas of flowers [38,39]. Therefore, elongated mouthparts may be an indicator of nectar consumption in flower-visiting insects. In beetles, proboscis formations are rare and have been found only in a few Meloidea [40]. However, based on feeding behaviour and without an analysis of gut contents, it can be difficult to interpret mouthpart structure. The morphological adaptions of the mouthparts to a specific diet possibly suggest modification of the gut in beetles that are specialised to process pollen and/or nectar in contrast to floral tissue feeders.

### 4.2. Alimentary Tract Structure and Lengths in Relation to Diet

The general morphology of the alimentary tract in monkey beetles as well as its subdivision into functional regions are similar to other beetles, except the lack of outgrowths or diverticula [15,16,17,18,19,20,21,22,23]. The proventriculus of many insects, including Coleoptera, is well developed and forms a grinding apparatus consisting of strong cuticle plates [14]. In monkey beetles, the proventriculus with its four folds and the lack of sclerotized structures is likely of lesser importance for food processing compared to other insect species. The discovery of a rudimentary proventriculus in monkey beetles contradicts previous findings [11]. A micromorphological investigation using semithin sectioning techniques of the proventriculus and its muscular structures in monkey beetles could clarify the role of the proventriculus during digestion. Unlike other nectar-feeding insects, nectar-feeding monkey beetles lack nectar crops or suction pumps in the foregut (reviewed in [37]).

Across the different monkey beetles there were no clear morphological features in the gut that appeared to be related to a particular food preference. A notable feature was the length of the foregut. Although based on a rather small sample size, it showed a noticeable trend, increasing in length in pollen- and nectar-feeding species, compared to floral tissue-feeding species. In monkey beetles, the longer foregut in nectar and pollen feeders could be a consequence of the greater relative lengths of head and thorax in comparison to tissue-feeding species. Likewise, the slight difference in head and mouthpart orientation, which are more anteriorly positioned in nectar/pollen tissue feeders than in floral tissue feeders [7,41], could influence the length of the pharynx and oesophagus. This difference may increase the ability to consume nectar and pollen from deeper corollas. These length differences of the foregut might also increase the storage capacity of the gut region. The longer foregut in pollen and nectar feeders could represent an adaptation to fluid or pollen diets; however, this would require confirmation with a larger sample size and experimental studies. When comparing females and males, a trend to longer midguts in female monkey beetles was noticed. In other beetle species, different lengths of the midgut have been observed in relation to diet; for example, carnivorous beetles have shorter midguts than herbivorous beetles [15]. The length of the alimentary tract in monkey beetles was greatly influenced by the number of midgut loops present in the beetles’ abdomens. While all monkey beetle species had similarly arranged loops, there were intraspecific and interspecific differences, as well as visual differences between the sexes.

### 4.3. Gut Content

In terms of gut contents, pollen was easily identified and plant fibres indicated uptake of petals, ovules, or entire florets of inflorescences. However, dissection could not determine whether a beetle had consumed nectar. The gut content was often ambiguous, and nectar could not be confirmed due to liquid preservation issues. Only *Anisonyx* species and *P. crassipes* were classified as putative nectar feeders based on mouthpart morphology and the fact that less floral tissue and pollen were found in their midguts when compared to presumed floral tissue and pollen feeders.

Several mechanisms for pollen digestion occur in insects [11,42,43,44,45,46]. In monkey beetles, undamaged pollen was found in the foregut up to the anterior midgut. A loss of pollen content and change of pollen colour was noticed in the posterior region of the midgut and in the hindgut. We conclude that enzymes penetrated the exine or passed through pollen pores and digested the protoplasmic content inside. The products of this digestion may extrude via the same pores out of the pollen grain resulting in empty or collapsed pollen inside the hindgut of monkey beetles [11].

Observations of mouthpart movements in monkey beetles [11] and European flower-visiting Cetoniinae [10] indicate that the sweeping movements of the maxillae take up nectar and pollen grains simultaneously. Unlike other studies of nectar-feeding monkey beetles [7], this study found no coloured fluid in the dissected specimens of *Anisonyx*. However, *Anisonyx* species are non-embedding and visit flowers that produce nectar, which suggests nectar feeding [29]. However, the presence of nectar in the digestive tract alone does not definitively prove that a species is specialized to consumes only nectar. Nectar is available in many flowering plants and could be used by beetles of different feeding guilds for hydration or quick energy uptake.

Based on feeding observations, it was expected that the studied beetles would feed from a specific food type and possess adequately adapted mouthparts. However, the mismatching of the gut content and mouthpart adaptations to a different food type did occur in several species. We suggest that species like *P. crassipes* are, based on our limited data, generalists and cannot be assigned a specific feeding preference. Mouthparts further suggest a distinction between solid and fluid food, given the similarities in form and structure between observed pollen- and floral-tissue-feeding monkey beetles, when compared to the mouthparts of nectar feeders, while the comparison of the gut morphology could not reveal unambiguous/specialized features in the studied monkey beetles.

## 5. Conclusions

This study on flower-visiting beetles is the first attempt to investigate whether diet preferences based on feeding observation and gut content are reflected in the morphology of mouthparts and the alimentary tract. Our results are based on mostly endemic species collected in the field and, consequently, rather few specimens of each species have been removed from the environment. Although this study is based on a small dataset concerning individuals per species, it revealed intriguing trends regarding gut length and feeding preference. Mouthparts of monkey beetles exhibit micro-morphological adaptations that can be interpreted in the context of solid (floral tissue, pollen) and/or fluid (nectar) feeding. In contrast, the alimentary tract lacks definitive morphological adaptations, and only the relative length of the foregut seems to be related to a nectar/pollen diet in some monkey beetles. Gut content did not correspond to mouthpart morphology across many species, and the assignment of feeding guild is not reflected in all studied beetles unambiguously.

The micro-CT technique turned out to be an appropriate method to study the morphology of the alimentary tract and gut content of Hopliini. Although it proved to be suitable for detailed investigations of a few species, it is rather time consuming when used for a larger range of species. Morphological studies of the digestive tract of flower-visiting insects are rare [47], although this method possess a high potential to study internal morphology in relation to feeding ecology of other various flower visiting insects.

Finally, although constrained by small sample size, our study highlights morphological variation in mouthparts and alimentary tract morphology among monkey beetles. These patterns suggest potential links to diet, but more comprehensive sampling and experimental analyses will be required to test these ecomorphological relationships.

## Figures and Tables

**Table 1:** Studied monkey beetle species with their respective number of specimens, males and females; total number of specimens (*n* = 23).

Species (*n* = 10)	Number of Specimens	Males	Females
*Anisochelus inornatus* Burmeister, 1844	2	1	1
*Clania glenlyonensis* Dombrow, 1997	2	1	1
*Heterochelus detritus* Burmeister, 1844	3	2	1
*Scelophysa strandfonteinensis* Dombrow, 1999	2	1	1
*Anisonyx ditus* Péringuey, 1902	2	1	1
*Anisonyx ursus* Fabricius, 1775	2	0	2
*Pachycnema crassipes* (Fabricius, 1775)	2	1	1
*Beckhoplia gifbergensis* Dombrow, 2020	2	0	2
*Denticnema squamosum* (Burmeister, 1844)	4	2	2
*Lepisia colvillei* Dombrow, 2006	2	1	1
Total number	23	10	13

**Table 2:** Monkey beetles (n = 10) with their respective predicted feeding group, gut content and mouthpart food type; floral tissue (FT), nectar (N), pollen (P), data not available (na).

Species	According to Observations	Gut Content	Mouthpart Food Type
*A. inornatus*	P	FT	FT
*C. glenlyonensis*	P	FT	FT
*H. detritus*	FT	na	FT
*S. strandfonteinensis*	FT	FT	FT
*A. ditus*	N	N	N
*A. ursus*	N	N	N
*P. crassipes*	FT	P	N
*B. gifbergensis*	P	P	P
*D. squamosum*	FT	na	P
*L. colvillei*	P	P	P

**Table 3:** Measurements of studied specimens (*n* = 23); head length (HL), body length (BL), thorax length (TL), total gut length (TGL), foregut length (FGL), midgut length (MGL), hindgut length (HGL).

Species	Sex	BL (mm)	HL (mm)	HL:BL	TL (mm)	TL:BL	TGL (mm)	TGL:BL	FL (mm)	FGL:BL	ML (mm)	MGL:BL	HL (mm)	HGL:BL
*Anisochelus* *inornatus*	f	7.18	1.58	0.22	2.85	0.40	17.51	2.44	1.57	0.22	8.93	1.24	7.02	0.98
	m	5.98	1.31	0.22	3.07	0.51	10.86	1.82	1.38	0.23	4.42	0.74	5.06	0.85
*Clania* *glenlyonensis*	f	8.82	1.99	0.23	3.56	0.40	34.92	3.96	1.69	0.19	22.27	2.52	10.97	1.24
	m	8.66	2.00	0.23	3.72	0.43	22.86	2.64	1.53	0.18	10.47	1.21	10.86	1.25
*Heterochelus* *detritus*	f	9.60	1.80	0.19	4.23	0.44	22.33	2.33	2.28	0.24	11.82	1.23	8.26	0.86
	m	8.74	1.91	0.22	4.32	0.49	21.52	2.46	2.29	0.26	9.43	1.08	9.80	1.12
	m	8.57	1.80	0.21	4.09	0.48	21.19	2.47	2.02	0.24	9.16	1.07	10.01	1.17
*Scelophysa* *strandfonteinensis*	f	10.27	2.18	0.21	4.10	0.40	44.36	4.32	2.39	0.23	27.29	2.66	14.67	1.43
	m	9.82	2.10	0.21	4.17	0.42	30.54	3.11	1.86	0.19	17.21	1.75	11.46	1.17
*Anisonyx ditus*	f	8.01	2.28	0.28	3.42	0.43	25.47	3.18	2.36	0.29	13.88	1.73	9.23	1.15
	m	8.16	2.41	0.30	3.17	0.39	17.21	2.11	2.29	0.28	7.56	0.93	7.35	0.90
*Anisonyx ursus*	f	10.38	2.73	0.26	4.11	0.40	29.46	2.84	2.89	0.28	14.46	1.39	12.09	1.17
	f	10.05	2.52	0.25	3.73	0.37	31.45	3.13	2.91	0.29	16.90	1.68	11.63	1.16
*Pachycnema* *crassipes*	f	8.57	2.05	0.24	3.37	0.39	27.01	3.15	2.10	0.24	15.14	1.77	9.76	1.14
	m	8.78	2.12	0.24	3.76	0.43	25.16	2.87	2.15	0.24	11.74	1.34	11.28	1.29
*Beckhoplia* *gifbergensis*	f	7.04	1.48	0.21	2.77	0.39	17.06	2.42	1.59	0.29	7.18	1.02	8.29	1.18
	f	8.23	1.66	0.20	3.09	0.38	16.21	1.97	1.83	0.28	6.20	0.75	8.18	0.99
*Denticnema* *squamosum*	f	10.18	1.96	0.19	4.05	0.40	24.10	2.37	2.45	0.28	12.68	1.25	8.97	0.88
	f	10.62	1.92	0.18	4.20	0.40	28.53	2.69	2.69	0.24	13.73	1.29	12.11	1.14
	m	8.58	1.86	0.22	3.98	0.46	22.44	2.62	2.05	0.29	10.51	1.23	10.02	1.17
	m	9.63	1.95	0.20	4.35	0.45	19.83	2.06	1.94	0.24	8.39	0.87	9.49	0.99
*Lepisia colvillei*	f	9.51	2.10	0.22	3.61	0.38	36.20	3.81	1.92	0.20	23.63	2.49	10.66	1.12
	m	8.92	2.11	0.24	3.71	0.42	28.77	3.23	1.70	0.19	15.61	1.75	11.47	1.29

## Data Availability

The data presented in this study are available on request from the corresponding author due to privacy reasons.

## Abbreviations

The following abbreviations are used in this manuscript:

CT	Computer tomography
FAA	Formalin-aceto-alcohol
I2E	1% iodine metal dissolved in 100% ethanol
